# Human Amniotic Fluid Stem Cell-Derived Exosomes as a Novel Cell-Free Therapy for Cutaneous Regeneration

**DOI:** 10.3389/fcell.2021.685873

**Published:** 2021-06-21

**Authors:** Yan Zhang, Jiaqing Yan, Yanhong Liu, Zhenyu Chen, Xiheng Li, Liang Tang, Jiang Li, Mengna Duan, Guokun Zhang

**Affiliations:** ^1^Hospital of Stomatology, Jilin University, Changchun, China; ^2^Jilin Provincial Laboratory of Biomedical Engineering, Jilin University, Changchun, China; ^3^Center for Reproductive Medicine, Center for Prenatal Diagnosis, The First Hospital of Jilin University, Changchun, China; ^4^Chengnan Branch, Foshan Stomatology Hospital, School of Stomatology and Medicine, Foshan University, Foshan, China; ^5^Affiliated Stomatology Hospital, Guangzhou Medical University, Guangzhou, China; ^6^Institute of Antler Science and Product Technology, Changchun Sci-Tech University, Changchun, China; ^7^Institute of Special Animal and Plant Sciences, Chinese Academy of Agricultural Sciences, Changchun, China

**Keywords:** human amniotic fluid stem cells, exosomes, miRNA, scarring, transforming growth factor, wound healing

## Abstract

Adult wound healing often results in fibrotic scarring that is caused by myofibroblast aggregation. Human amniotic fluid stem cells (hAFSCs) exhibit significantly anti-fibrotic scarring properties during wound healing. However, it is little known whether hAFSCs directly or indirectly (paracrine) contribute to this process. Using the full-thickness skin-wounded rats, we investigated the therapeutic potential of hAFSC-derived exosomes (hAFSC-exo). Our results showed that hAFSC-exo accelerated the wound healing rate and improved the regeneration of hair follicles, nerves, and vessels, as well as increased proliferation of cutaneous cells and the natural distribution of collagen during wound healing. Additionally, hAFSC-exo suppressed the excessive aggregation of myofibroblasts and the extracellular matrix. We identified several miRNAs, including let-7-5p, miR-22-3p, miR-27a-3p, miR-21-5p, and miR-23a-3p, that were presented in hAFSC-exo. The functional analysis demonstrated that these hAFSC-exo-miRNAs contribute to the inhibition of the transforming growth factor-β (TGF-β) signaling pathway by targeting the TGF-β receptor type I (TGF-βR1) and TGF-β receptor type II (TGF-βR2). The reduction of TGF-βR1 and TGF-βR2 expression induced by hAFSC-exo was also confirmed in the healing tissue. Finally, using mimics of miRNAs, we found that hAFSC-exo-miRNAs were essential for myofibroblast suppression during the TGF-β1-induced human dermal fibroblast-to-myofibroblast transition *in vitro*. In summary, this study is the first to show that exosomal miRNAs used in hAFSC-based therapy inhibit myofibroblast differentiation. Our study suggests that hAFSC-exo may represent a strategic tool for suppressing fibrotic scarring during wound healing.

## Introduction

The skin provides a natural and significant protective barrier for the human body. However, severe disabilities or even death may occur if its integrity is damaged (Coughlin and Taïeb, 2014; Dyring-Andersen et al., 2020). The ultimate goal of wound therapy is to rapidly close and shape the lesion and functionally restore the skin (Reinke and Sorg, 2012; Sorg et al., 2017). Previous studies showed that cutaneous wound healing is a complex process involving three overlapping stages: inflammation, tissue formation, and tissue remodeling (Reinke and Sorg, 2012; Sorg et al., 2017). During the process of wound re-epithelization, the appearance of myofibroblasts is indispensable. However, myofibroblasts generally promote aberrant recruitment and maintenance excessively during wound healing in adults, leading to fibrotic scarring (Wynn and Ramalingam, 2012; Rippa et al., 2019). Diverse sources of myofibroblasts have been proposed, including resident fibroblasts, epithelial and endothelial cells (*via* epithelial or endothelio-mesenchymal transition), circulating fibrocytes derived from bone marrow, and pericytes (Rippa et al., 2019). It is reported that more than 50% of myofibroblasts may come from the differentiation of resident fibroblasts induced by the transforming growth factor-β (TGF-β) family (Hinz, 2016; Pardali et al., 2017; Rosenbloom et al., 2017). TGF-β binds to TGF-β receptors that phosphorylate various Smad proteins. Phosphorylated Smad activates downstream target genes including α-smooth muscle actin (α-SMA) and collagen I, which promote myofibroblast differentiation (Fang et al., 2016; Hinz, 2016). Interfering with the activity of the TGF-β receptor (TGF-βR) disrupts the function of TGF-β and suppresses myofibroblast differentiation and over-aggregation. This reduces excessive fibrotic scarring during wound healing.

Recent studies have indicated that stem cell therapy represents a novel approach for reducing fibrotic scarring during wound healing (Fang et al., 2016; Duan et al., 2020; Rong et al., 2020). Stem cells exhibit several favorable characteristics, including relatively easy expansion *in vitro*, migration to the injury site, and an ability to differentiate into the cell types required for tissue repair (Abdelwahid et al., 2016; Han et al., 2019; Duan et al., 2020; Rong et al., 2020). However, the successful use of adult stem cells is limited because they retain epigenetic alterations even after reprogramming (Han et al., 2019; Castelli et al., 2020). Fetal stem cells may overcome this limitation, especially those derived from the amniotic fluid collected during a cesarean section, third trimester amnio-reduction, or routine amniocentesis (Roubelakis et al., 2012; Balbi et al., 2017; Loukogeorgakis and De Coppi, 2017; Castelli et al., 2020). Previous studies have shown that human amniotic fluid stem cells (hAFSCs) can accelerate cutaneous wound healing with less fibrotic scarring, similar to fetal wound healing (Skardal et al., 2012; Yang et al., 2013; Jun et al., 2014; Fukutake et al., 2019). Nevertheless, only a small number of stem cells can remain at the injured site following stem cell therapy to directly participate in the regenerated tissue formation (Fang et al., 2016; Taverna et al., 2017; Sriramulu et al., 2018; Park et al., 2019; Duan et al., 2020). We hypothesize that the powerful paracrine capacity of hAFSCs may be the main contributor to its therapeutic efficacy.

Exosomes are small vesicles secreted by cells in the range of 30–150 nm that contain paracrine factors or “cargo,” such as miRNAs, DNA, proteins, saccharides, and lipids (Batrakova and Kim, 2015; Pegtel and Gould, 2019; Duan et al., 2020). Exosomes contribute to cell-to-cell communication, biomarker secretion, biological information storage, and tissue regeneration processes (Batrakova and Kim, 2015; Pegtel and Gould, 2019).

In this study, we evaluated the effects of hAFSC-derived exosomes (hAFSC-exo) on anti-fibrotic scarring during wound healing using the full-thickness skin-wounded rat model. Additionally, we identified five specific miRNAs transported by hAFSC-exo as essential factors that suppress fibroblast differentiation into myofibroblasts by inhibiting TGF-βR expression. Our results indicate that hAFSC-exo treatment represents a promising tool for suppressing fibrotic scarring during cutaneous wound healing.

## Materials and Methods

### Cell Expansion

Human amniotic fluid stem cells were from leftover samples of amniotic fluid obtained *via* amniocentesis upon written informed consent, as previously described (Balbi et al., 2017). All procedures were performed in compliance with the Helsinki Declaration. hAFSCs were characterized by surface marker profiling (CD34, CD45, CD73, CD90, and CD105) *via* immunofluorescence (IF) staining. The multipotency of hAFSCs was detected *via* inducing chondrogenic (Alcian Blue staining), osteogenic (Alizarin Red S staining), and adipogenic (Oil Red O staining) differentiation. The P2-P5 hAFSCs were used for experiments.

The human dermal fibroblasts (HDFs) were a gift from Dr. Shi (China-Japan Union Hospital, Jilin University), which were isolated from the dermal tissue of the juvenile foreskin. The cells (third to eighth passage) were cultured in Dulbecco’s Modified Eagle Medium (DMEM; Gibco, United States) containing 10% fetal bovine serum (FBS; HyClone, China) in a humidified 5% (v/v) CO_2_ atmosphere at 37°C.

### Exosome Isolation

The hAFSCs were grown to 80% confluency in DMEM containing 10% FBS, then replaced with serum-free medium (Biological Industries, Israel) and cultured for 48 h. The culture suspension was collected and centrifuged as follows: 2,000*g* for 10 min (removing dead cells) → 10,000*g* for 30 min (removing debris) → 100,000*g* for 70 min (collecting exosomes). The exosomes were then washed three times with PBS. The protein concentration of hAFSC-exo was measured using the bicinchoninic acid assay (Solarbio, China). The hAFSC-exo product was identified using transmission electron microscopy, NanoSight NS300 (Malvern Instruments, United Kingdom), and exosomal markers CD9, CD63, and TSG101 (Beyotime, China) using western blot analysis. The exosomes were stored at −80°C for subsequent studies.

### Cell Treatment

Human dermal fibroblasts were cultured in 24-well plates (50,000 cells/well; 500 μl), add DMEM + 10% FBS (+TGF-β1, 25 ng/ml) culture for 48 h to induce myofibroblast differentiation *in vitro*. hAFSC-exo (25 ng/ml) was added synchronously, and the intervention effect on the treated cells was evaluated. The intervention effect on the treated cells of miR-21-5p, miR-23a-3p, let-7-5p, miR-22-3p, or miR-27a-3p was also evaluated using mimics (50 nM); Lipofectamine 3000 (Thermo Fisher Scientific, United States) was used for the transfection of mimics; a nonsense sequence was used as the negative control (NC). The expression of α-SMA, TGF-βR type I (TGF-βR1), and TGF-βR type II (TGF-βR2) was measured by quantitative real-time polymerase chain reaction (qRT-PCR), IF staining, and western blot analysis.

### Cutaneous-Wounded Model Establishment and Treatment

Thirty 8-week-old SD rats (female, 200 g) were selected and fed under standard feeding conditions. The experimental protocols and procedures involving the rats were approved by the Animal Experimental Ethics Committee of Jilin University (Approval No.: SY201902011). The rats were anesthetized with 3% pentobarbital sodium (30 mg/kg), and the back hair was cut with scissors. Then a circular hole (12 mm in diameter), full-thickness skin excisional wounds were made on the shaved skin. The rats were randomly divided into three groups (*n* = 10/group), and different agents (CTRL: PBS; hAFSC: 2 × 10^6^ cells; and hAFSC-exo: 20 μg exosomes) were injected (100 μl) subcutaneously around the wound once every 7 days for 28 days. Photos were taken each 7 days to record the extent of wound healing. The healing wound tissues of the rats were collected for future study after the rats were euthanized with 3% pentobarbital sodium (90 mg/kg).

### Histology and IF Staining

Healing tissue skin was fixed with 4% paraformaldehyde for 48 h and embedded in paraffin. In accordance with the manufacturer’s instructions, 4.0-μm sections were prepared and stained with H&E (Meilune, China) and Masson (Solarbio, China). For IF staining, fixed-cells or rehydrated antigen-repaired paraffin sections were incubated with specific primary antibodies (PBS incubation as a control) and stained with Cy3- and AF 488-labeled secondary antibodies (Thermo Fisher Scientific, United States). Supplementary Table 1 lists the primary antibodies. Nuclear staining was performed with DAPI (Beyotime, China). The images of the stained sections were obtained by microscopy (Precipoint M8 Digital Imaging Scanning System, Germany; and EVOS M5000 Imaging System, United States). The number of hair follicles and the epidermal thickness were analyzed according to H&E staining; three fields (20×) containing the epidermis in the healed skin were randomly selected, then the hair follicles were counted, and the thickness of the epidermis was quantified based on the scale bar. The collagen fibers were quantified using Image-Pro Plus software. Firstly, we counted the blue area (collagen fibers) in the micrograph and then calculated its ratio to the total area of the micrograph.

### qRT-PCR Analysis

Total RNA was isolated from tissues, cells, or hAFSC-exo using TRIzol Reagent (Sigma, United States) and reverse-transcribed into cDNA using the PrimeScript RT reagent kit (Takara, Japan). qRT-PCR was performed on an ABI 9700 Detection System (Thermo Fisher Scientific, United States). The relative expression levels of the target genes were calculated using the 2^–ΔΔ*Ct*^ method. GAPDH and U6 were used as controls for the mRNA and miRNA reactions, respectively. Supplementary Table 2 lists the primers. All experiments were repeated three times.

### Western Blot Analysis

Total proteins were extracted from each sample using RIPA reagent (Invitrogen, United States) and then separated *via* polyacrylamide SDS gel and electrophoretically transferred onto polyvinylidene fluoride membranes (Millipore, MA, United States). The membranes were incubated with the primary antibodies (Supplementary Table 1) and then the secondary antibody. Levels of proteins were determined using an ECL system.

### Statistical Analysis

Results were shown as the mean ± SD (*n* = 3). Statistical analysis was conducted using GraphPad Prism 8 software, and significant differences were evaluated using a one-way analysis of variance. *P* < 0.05 was considered statistically significant.

## Results

### hAFSC-exo Improve the Wound Healing Rate and Regeneration Ability in Full-Thickness Cutaneous-Wounded Rats

**FIGURE 1:**
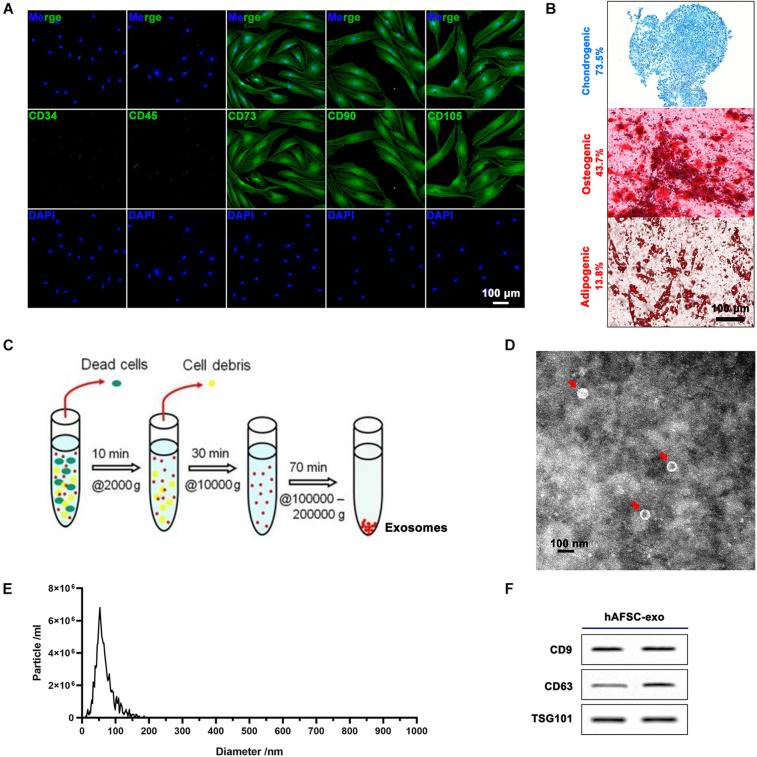
Characterization of hAFSCs and hAFSC-exo. **(A)** IF staining of cell surface markers CD34, CD45, CD73, CD90, and CD105; scale bar = 100 μm. **(B)** Osteogenic (Alizarin Red S staining), chondrogenic (Alcian Blue staining), or adipogenic (Oil Red O staining) differentiation induction of hAFSCs; scale bar = 100 μm. **(C)** Schematic representation of exosome isolation. hAFSC-exo were isolated from hAFSC supernatant using high-speed centrifugation. **(D)** Morphological characteristics of hAFSC-exo were imaged using transmission electron microscopy (scale bar = 100 nm). **(E)** hAFSC-exo particle size was measured using NanoSight. **(F)** Protein expression of exosome markers of hAFSC-exo, including CD9, CD63, and TSG101, was detected using western blot analysis. hAFSC, human amniotic fluid stem cells; hAFSC-exo, human amniotic fluid stem cell-derived exosomes.

Human amniotic fluid stem cells expressed cell surface markers, including CD73, CD90, and CD105, and were negative for CD34 and CD45 (Figure 1A). Further hAFSCs could be induced to chondrogenic, osteogenic, and adipogenic differentiation with a positive rate of 73.5, 43.7, and 13.8%, respectively (Figure 1B). Then, we prepared hAFSC-exo by gradient centrifugation (Figure 1C) and characterized the product. The results indicated that hAFSC-exo exhibited a round-shaped morphology (Figure 1D) with an average diameter of 70 ± 20 nm (Figure 1E) using a transmission electron microscope and NanoSight. hAFSC-exo also expressed exosomal markers, including CD9, CD63, and TSG101, as determined by western blot analysis (Figure 1F).

**FIGURE 2:**
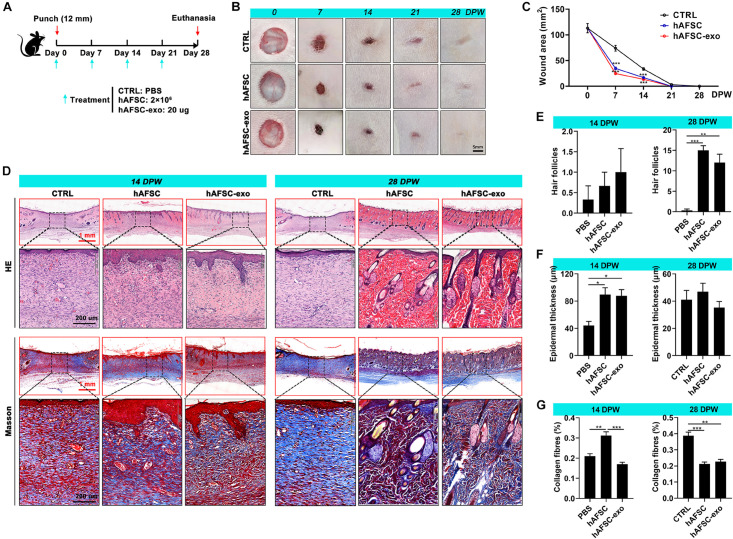
hAFSC-exo accelerated the wound healing rate and improved the regeneration quality in the full-thickness cutaneous-wounded rats. **(A)** Schematic representation of the experimental design. The full-thickness cutaneous-wounded model (12 mm diameter) was established on the shaved skin of the dorsal region. The rats were then administered treatments by local injection every 7 days: CTRL (PBS), hAFSC (2 × 10^6^ cells), and hAFSC-exo (20 μg). **(B)** Morphological changes in the wound healing process (scale bar = 5 mm). **(C)** Changes in wound area during wound healing. **(D)** H&E and Masson staining of the healing tissues; scale bar (red) = 1 mm; scale bar (black) = 200 μm. **(E–G)** Number of hair follicles/field (20×), epidermal thickness, and percentage of collagen fibers (blue area in Masson staining) in the healing tissue according to histology. *P < 0.05; **P < 0.01; ***P < 0.001; mean ± SD; *n* = 5. CTRL, control; DPW, days post-wounding.

To determine whether treatment with hAFSC-exo improves wound healing, we evaluated the therapeutic effects of hAFSC-exo using a full-thickness cutaneous-wounded rat model, hAFSCs were used as the positive control (Figure 2A). Expectedly, we observed no significant differences in healing rate and quality in the hAFSC-exo and hAFSC groups, except for the collagen fiber level at 15 days post-wounding (DPW). However, compared with the PBS (CTRL), hAFSC-exo accelerated wound healing (Figures 2B,C). Especially early (at 7 DPW), the difference in wound area between the CTRL group and the AFSC-exo group was the most significant. At 28 DPW, after the wounds were repaired, the hAFSC-exo group exhibited a smoother wound edge than the CTRL group (Figure 2B). Furthermore, we performed a histological evaluation of the healing tissues using H&E and Masson staining. The results showed that the healing skin in the hAFSC-exo group exhibited a thicker epidermal layer at 14 DPW and more hair follicles at 28 DPW compared with that in the CTRL group (Figures 2D–F). The healing skin in the hAFSC-exo group exhibited a lower level of collagen fibers, which was more neatly arranged, compared with that in the CTRL group at 28 DPW (Figures 2D,G). These results suggest that hAFSC-exo can accelerate the wound healing rate, improve hair follicle regeneration, and reduce collagen fiber deposition.

### hAFSC-exo Promote Nerve and Vessel Restoration of Healing Skin in Rats

**FIGURE 3:**
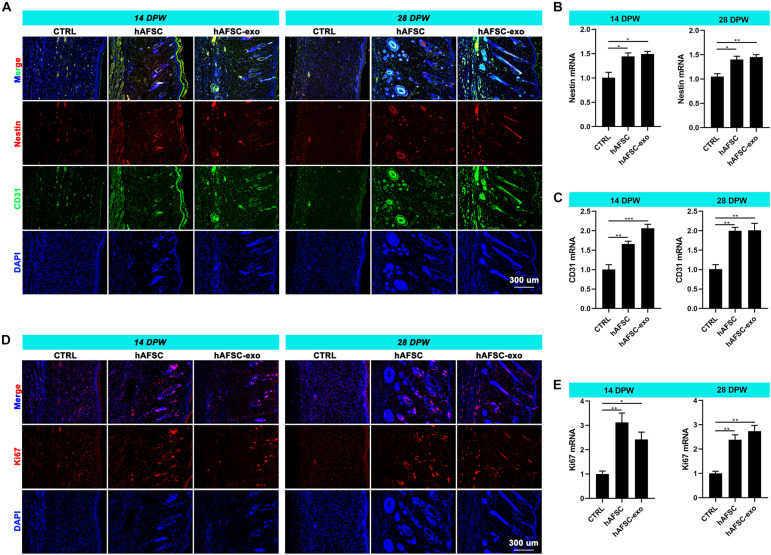
hAFSC-exo promotes the regeneration of nerves and vessels during wound healing in rats. **(A–C)** IF staining and qRT-PCR analysis of Nestin (nerve) and CD31 (vessel) in the healed tissues. **(D,E)** IF staining and qRT-PCR analysis of Ki67 in the healed tissues. Scale bar = 300 μm; *P < 0.05; **P < 0.01; ***P < 0.001; mean ± SD; *n* = 5. DAPI, 4′,6-Diamidino-2-phenylindole; IF, immunofluorescence.

The reconstruction of nerves and vessels is essential for recovering damaged skin function (Duan et al., 2020). Therefore, we investigated the effects of hAFSC-exo on nerve and vessel regeneration of wounded skin using IF staining and qRT-PCR analysis. The results showed that the healing skin of the hAFSC-exo group exhibited significantly more Nestin (a marker of nerves) (Duan et al., 2020) and CD31 (a marker of vessels) (Duan et al., 2020) compared with that in the CTRL group (Figures 3A–C). Furthermore, the IF (Ki67) staining revealed that the proliferation rate of cutaneous cells of healing skin in the hAFSC-exo group was faster than that in the CTRL group (Figures 3D,E). These results suggest that hAFSC-exo can promote the reconstruction of nerves and vessels and increase the proliferation of cutaneous cells during wound healing.

### hAFSC-exo Suppress Myofibroblast Aggregation During Wound Healing in Rats

**FIGURE 4:**
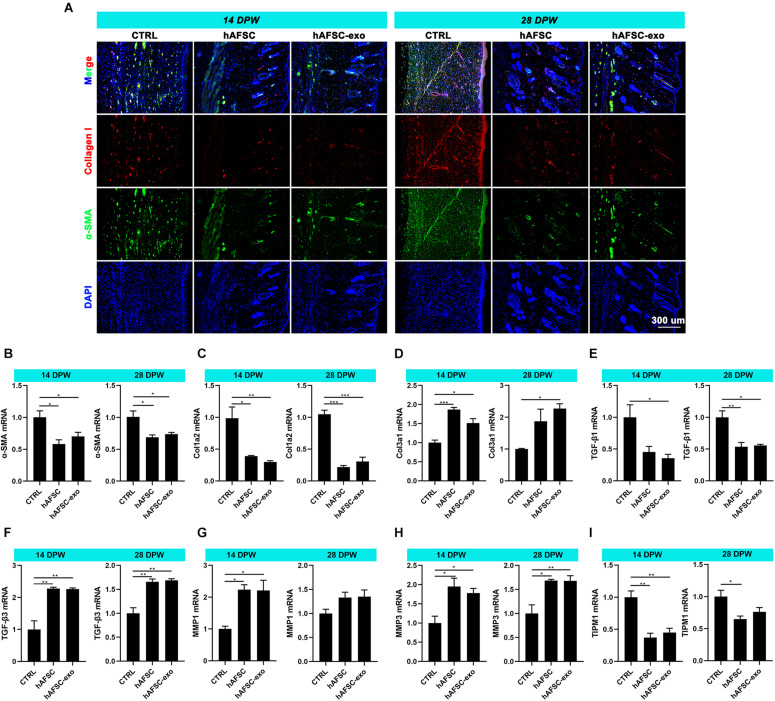
hAFSC-exo suppress myofibroblast aggregation on wound healing in rats. **(A)** IF staining of α-SMA and collagen I in the healed tissues (scale bar = 300 μm). **(B–I)** mRNA levels of α-SMA, Col1a2, Col3a1, TGF-β1, TGF-β3, MMP1, MMP3, and TIMP1 in the healed tissues. *P < 0.05; **P < 0.01; ***P < 0.001; mean ± SD; *n* = 5. α-SMA, α-Smooth muscle actin; MMP1, matrix metalloproteinase 1; MMP3, matrix metalloproteinase 3; TGF-β1, transforming growth factor-β1; TGF-β3, transforming growth factor-β3; TIMP1, tissue Inhibitor of metalloproteinases 1.

Myofibroblasts generally aberrate recruitment and maintenance excessively during wound healing in adults, leading to fibrotic scarring (Reinke and Sorg, 2012; Sorg et al., 2017). However, scarless healing of fatal skin is not associated with the excessive accumulation of myofibroblasts (Colwell et al., 2003; Helmo et al., 2013). The IF staining results showed that the expression levels of α-SMA and collagen I in healing skin were significantly reduced in the hAFSC-exo group compared with those in the CTRL group (Figures 4A–C). We measured the expression levels of fetal cutaneous regeneration-related genes (Colwell et al., 2003; Helmo et al., 2013) in healing rat skin using qRT-PCR. The results indicated that compared with those of the CTRL group, the mRNA levels of Col3a1, TGF-β3, MMP1, and MMP3 of the hAFSC-exo group were significantly increased, whereas Col1a2, TGF-β1, and TIMP1 levels were significantly decreased (Figures 4C–I). The results suggest that hAFSC-exo reduce *in situ* myofibroblast formation, which resembled fatal wound healing.

### hAFSC-exo Suppress Myofibroblast Differentiation by Inhibiting the TGF-β Receptors

MiRNAs are the main components of functional RNA in exosomes (Batrakova and Kim, 2015; Fang et al., 2016; Pegtel and Gould, 2019; Duan et al., 2020). We analyzed the miRNAs in hAFSC-exo as reported by previous studies (Balbi et al., 2017; Sedrakyan et al., 2017; Castelli et al., 2020), and found 16 highly expressed miRNAs, including let-7-5p, miR-34-5p, miR-21-5p, miR-146-5p, miR-22-3p, miR-27-3p, miR-31-5p, miR-154-5p, miR-199-3p, miR-23-3p, miR-223-3p, miR-221-3p, miR-29-3p, miR-155-5p, miR-24-3p, and miR-28-5p (Supplementary Material 2).

**FIGURE 5:**
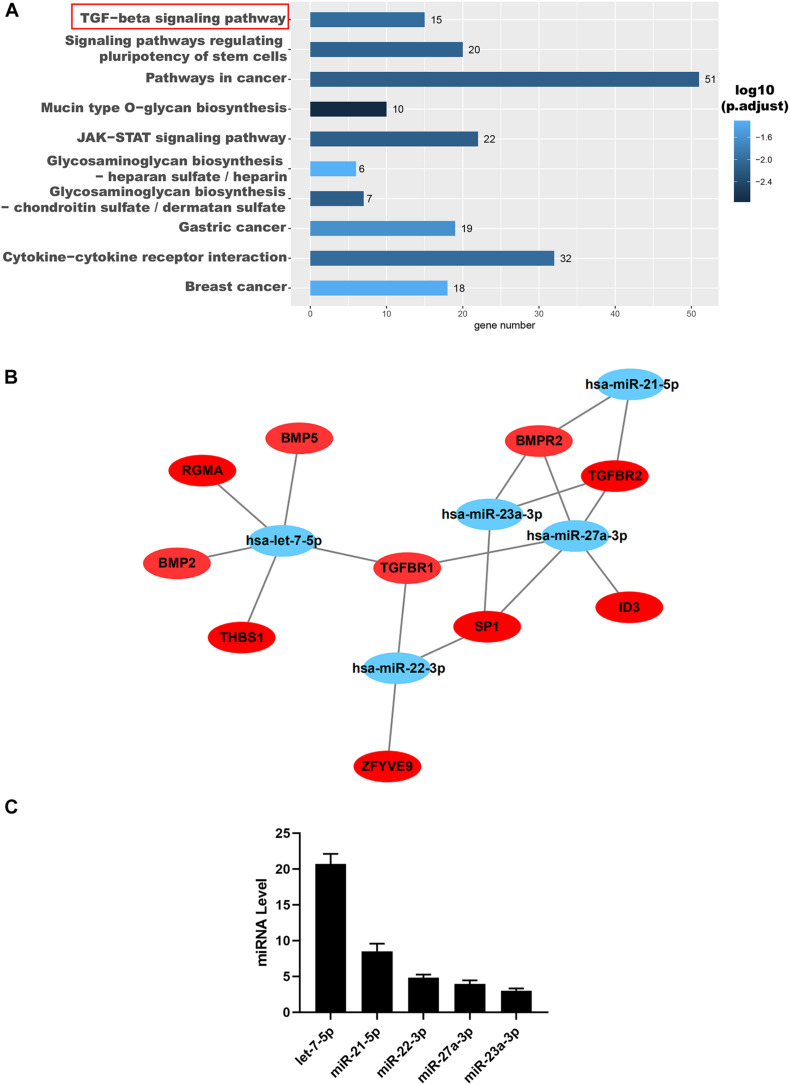
Identification and functional analysis of hAFSC-exo-miRNAs. **(A)** Target mRNA prediction of hAFSC-exo-miRNAs using Gene Ontology analysis; the TGF-β signaling pathway was highly related to myofibroblast formation. **(B)** The miRNA–mRNA interaction network of hAFSC-exo-miRNAs and their predicted targets. **(C)** Primary miRNAs in hAFSC-exo as measured by qRT-PCR analysis.

To further reveal the possible role of these miRNAs, we used TargetScan^1^ and Gene Ontology (GO) analysis to predict their target genes and function, respectively. The results indicated that these miRNAs were positively correlated with the TGF-β signaling pathway demonstrated by the GO analysis (Figure 5A). Interestingly, these miRNAs, including let-7-5p, miR-22-3p, miR-27a-3p, miR-21-5p, and miR-23a-3p, were all found to be directly targeted to TGF-βR1 and TGF-βR2 (Figure 5B). Then we confirmed that the miRNAs, including let-7-5p, miR-22-3p, miR-27a-3p, miR-21-5p, and miR-23a-3p, in hAFSC-exo were present using qRT-PCR analysis (Figure 5C).

**FIGURE 6:**
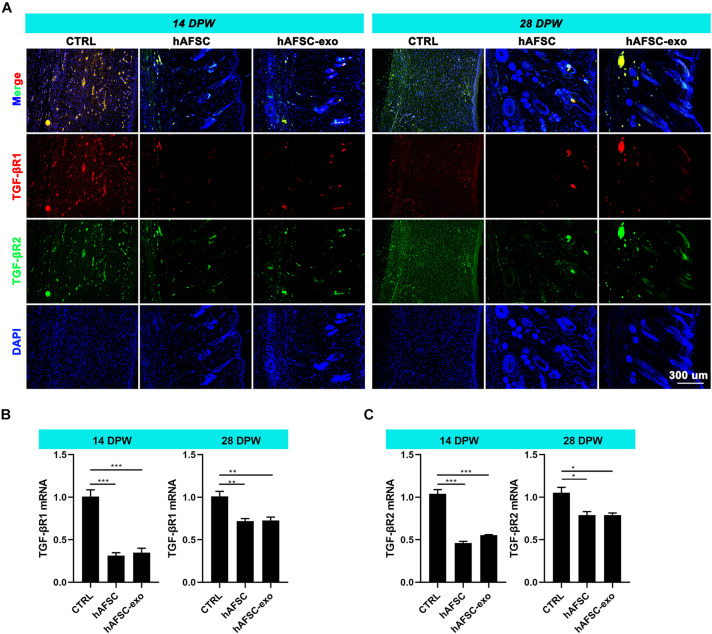
hAFSC-exo inhibited TGF-βR1 and TGF-βR2 expression on wound healing in rats. **(A)** IF staining of TGF-βR1 and TGF-βR2 in the healed tissues (scale bar = 300 μm). **(B,C)** mRNA levels of TGF-βR1 and TGF-βR2 in the healed tissues. Mean ± SD; *P < 0.05; **P < 0.01; ***P < 0.001; *n* = 5. TGF-βR1, transforming growth factor-β receptor type I, TGF-βR2, transforming growth factor-β receptor type II.

Next, we measured the expression levels of TGF-βR1 and TGF-βR2 in the healing skin of rats by IF staining and qRT-PCR analysis. The results showed that the expression levels of TGF-βR1 and TGF-βR2 were significantly reduced in the hAFSC-exo-treated group compared with those in the CTRL group (Figures 6A–C). These results suggest that hAFSC-exo-specific miRNAs may inhibit myofibroblast formation by inhibiting the activities of TGF-βR1 and TGF-βR2.

### hAFSC-exo Suppress the TGF-β1-Induced Human Dermal Fibroblast-to-Myofibroblast Transition

**FIGURE 7:**
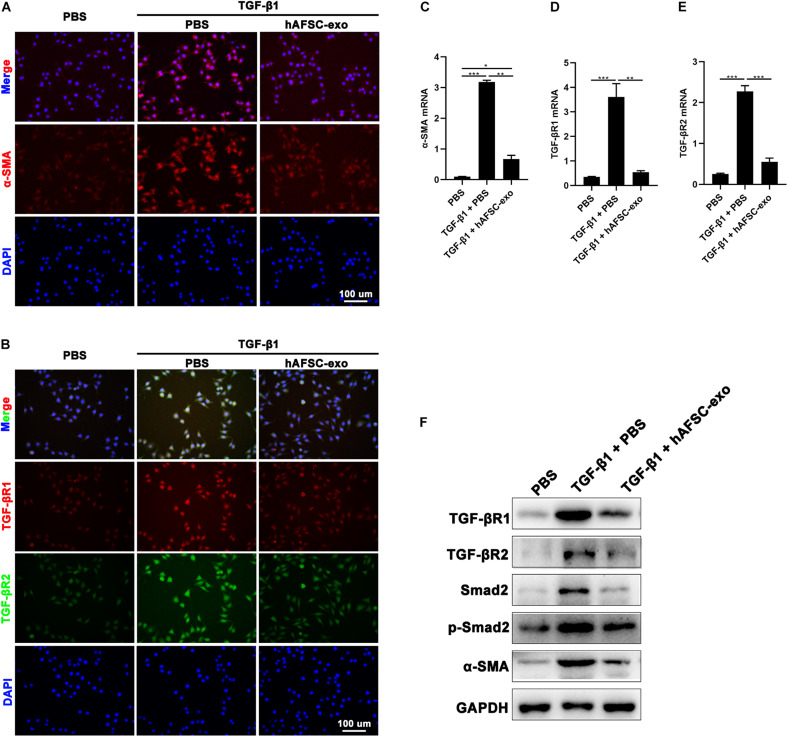
hAFSC-exo inhibited TGF-β-induced myofibroblast formation *in vitro*. HDFs were cultured for 48 h with TGF-β1 (25 ng/ml) to induce myofibroblast differentiation. Some of the cells were also treated with hAFSC-exo (10 ng/ml). **(A,B)** IF staining of α-SMA, TGF-βR1, and TGF-βR2 in HDFs (scale bar = 100 μm). **(C–E)** qRT-PCR analysis of α-SMA, TGF-βR1, and TGF-βR2 in HDFs. **(F)** Western blot analysis of TGF-βR1, TGF-βR2, Smad2, p-Smad2, and α-SMA in HDFs. Mean ± SD; *P < 0.05; **P < 0.01; ***P < 0.001; *n* = 3. HDFs, human dermal fibroblasts.

We cultured HDFs in the presence of TGF-β1 to stimulate myofibroblast formation *in vitro*. Cells were also treated with hAFSC-exo (10 ng/ml). We detected the expression of α-SMA in HDFs, and the results of IF staining and qRT-PCR analysis showed that hAFSC-exo treatment strongly inhibited the TGF-β1-induced high expression levels of α-SMA, TGF-βR1, and TGF-βR2 (Figure 7A–E). Furthermore, the expression levels of TGF-β signaling pathway related genes, including TGF-βR1, TGF-βR2, Smad2, p-Smad2, and α-SMA, were detected *via* western blot analysis. The results showed that the expression levels of those genes in TGF-β1-induced HDFs were all reduced by hAFSC-exo treatment (Figure 7F). These results suggest that hAFSC-exo inhibit TGF-β-induced myofibroblast formation *in vitro*.

### hAFSC-exo-Specific MiRNAs Target TGF-β Receptors to Suppress Myofibroblast Differentiation

**FIGURE 8:**
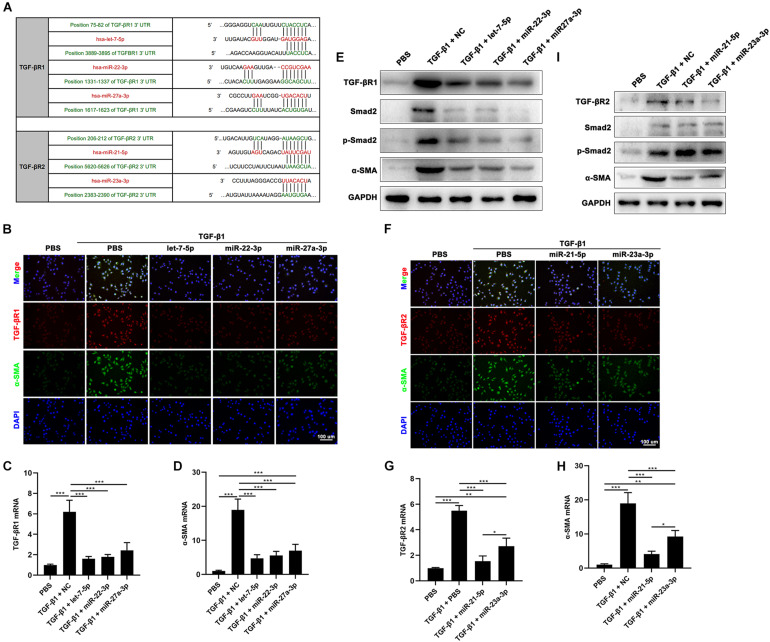
hAFSC-exo-miRNAs target the TGF-βR1 and TGF-βR2 to suppress myofibroblast formation. **(A)** List of predicted binding sites for hAFSC-exo-miRNAs and their targets. **(B–D)** Expression levels of α-SMA and TGF-βR1 in HDFs treated with specific mimics, including let-7-5p, miR-22-3p, and miR-27a-3p. **(E)** Western blot analysis of TGF-βR1, Smad2, p-Smad2, and α-SMA in HDFs treated with specific mimics, including let-7-5p, miR-22-3p, and miR-27a-3p. **(F–H)** Expression levels of α-SMA and TGF-βR2 in HDFs treated with specific mimics, including miR-21-5p and miR-23a-3p (scale bar = 100 μm). **(I)** Western blot analysis of TGF-βR2, Smad2, p-Smad2, and α-SMA in HDFs treated with specific mimics, including miR-21-5p and miR-23a-3p. Mean ± SD; *P < 0.05; **P < 0.01; ***P < 0.001; *n* = 3.

To investigate the function of hAFSC-exo-miRNAs, including let-7-5p, miR-22-3p, miR-27a-3p, miR-21-5p, and miR-23a-3p, we hypothesis that binding sites of these miRNAs on their putative targets (TGF-βR1 and TGF-βR2), can inhibit the TGF-β signaling pathway (Figure 8A). We used specific mimics of miRNA, to study the effects of related miRNAs on the expression α-SMA. Interestingly, let-7-5p, miR-22-3p, miR-27a-3p, miR-21-5p, and miR-23a-3p significantly reduced the expression of α-SMA (Figures 8B,D–F,H,I). Furthermore, we studied the effects of these miRNAs on the expression of TGF-βR1 and TGF-βR2. The results indicated that let-7-5p, miR-22-3p, and miR-27a-3p significantly inhibited the expression of TGF-βR1 (Figures 8B,C,E), whereas miR-21-5p and miR-23a-3p significantly inhibited the expression of TGF-βR2 (Figures 8F,G,I). Other related genes in the TGF-β signaling pathway, including Smad2 and p-Smad2, were also significantly reduced by these miRNAs (Figures 8E,I). Collectively, our results suggest that hAFSC-exo-specific miRNAs (let-7-5p, miR-22-3p, miR-27a-3p, miR-21-5p, and miR-23a-3p) inhibit the TGF-β signaling pathway and myofibroblast differentiation from fibroblasts by targeting TGF-βR1 and TGF-βR2 *in vitro*.

## Discussion

The standard for cutaneous repair includes the double recovery of structure and function. However, during adult wound healing, myofibroblasts are usually abnormally recruited and maintained, which results in fibrotic scarring (Reinke and Sorg, 2012; Sorg et al., 2017). Therefore, regulating myofibroblast formation may be a strategic way to prevent fibrotic scarring. Stem cell-based therapies have been developed as novel approaches for anti-fibrotic scarring in cutaneous wound healing (Ojeh et al., 2015; Fang et al., 2016; Kanji and Das, 2017; Rong et al., 2020). Particularly, hAFSCs are a type of fetal stem cell that is easily obtained within the scope of ethical regulations and exhibits superior effects in accelerating cutaneous wound healing and decreased fibrotic scarring (Skardal et al., 2012; Yang et al., 2013; Jun et al., 2014; Fukutake et al., 2019). However, few studies have been conducted to determine whether hAFSCs directly or indirectly, through paracrine components, contribute to these processes. This report confirms for the first time that hAFSCs inhibit the formation of myofibroblasts during wound healing, in part, through hAFSC-exo. Our findings provide new insight into the application of hAFSCs to prevent fibrotic scarring.

Naturally, stem cell or stem cell-derived exosome therapy exhibits structural and functional advantages by promoting cell proliferation at the injured site, stimulating neurogenesis and angiogenesis, and regulating the inflammatory response (Qu and Zhang, 2017; Sriramulu et al., 2018). Exosomes derived from adipose MSC (Hu et al., 2016), umbilical cord MSC (Fang et al., 2016), epidermal stem cells (Duan et al., 2020), and induced pluripotent stem cell-derived MSC (Zhang et al., 2015) improve cutaneous wound healing by delivering various functional RNAs, proteins, and other factors. High proliferation, low tumorigenicity and immunogenicity, and anti-inflammatory activity support hAFSCs as safe and effective donor cells for wound healing (Balbi et al., 2017; Castelli et al., 2020). The paracrine factors of hAFSCs have been reported to have the ability to stimulate cell proliferation and migration (Takov et al., 2020; Castelli et al., 2021). Furthermore, they have also been certified to modulate the redox unbalance due to unfavorable microenvironment positively (Ashour et al., 2016; Gatti et al., 2020). These abilities of hAFSC paracrine factors may have significant therapeutic functions for improving wound healing.

In this study, we evaluated the therapeutic effects of hAFSC-derived exosomes (hAFSC-exo) using a full-thickness cutaneous-wounded rat model. Our findings indicated that hAFSC-exo accelerated the wound healing rate and improved the regeneration levels of hair follicles, nerves, and vessels. Moreover, hAFSC-exo improved the natural distribution of collagen fibers and inhibited myofibroblast aggregation, thus suppressing fibrotic scarring.

This scarless wound healing may be achieved through the inhibition of TGF-β signaling pathway activation. It has been reported that TGF-β signaling is an essential regulatory factor in stimulating fibroblasts to differentiate into myofibroblasts (Hinz, 2016; Pardali et al., 2017). In this study, we observed that the expression of α-SMA in the hAFSC-exo group was lower than the CTRL group both *in vivo* and *in vitro*. α-SMA (induced by TGF-β signaling pathway) is considered to be a marker of myofibroblast differentiation (Hinz, 2016; Rippa et al., 2019). Scarring occurs to a certain extent physiologically, since myofibroblasts are inevitable in wound healing to close the wound. Hypertrophic scars occur when the myofibroblasts are not removed in later phases and persist with high activity (Hinz, 2016; Rippa et al., 2019). Therefore, intervention is required during wound healing to prevent myofibroblasts from accumulating rather than implementing remedial measures after fibrotic scarring. Besides the regenerative repair of cutaneous wounds to achieve scarless healing, rapid wound closure is also critical because wound closure is essential to block external environmental interference. Although the contractility of myofibroblasts is beneficial to wound closure in the early stage, the inhibition of hAFSC-exo on myofibroblast differentiation does not necessarily lead to a slowdown in wound healing. We found that hAFSC-exo can promote the proliferation and migration of skin cells, which may be essential for accelerating wound closure.

The therapeutic potential of exosomes is determined by the composition of the “cargo” they carry (Samanta et al., 2018). The “cargo” may include various growth factors and tissue regeneration factors released by stem cells (Samanta et al., 2018; Pegtel and Gould, 2019). In this study, we found that the binding effect of anti-fibrotic scarring may result from a set of TGF-βR-targeting miRNAs. We identified several highly abundant and specific miRNAs derived from hAFSC-exo, including let-7-5p, miR-22-3p, miR-27a-3p, miR-21-5p, and miR-23a-3p, all of which directly target TGF-βRs. Let-7-5p (Elliot et al., 2019), miR-22-3p (Hong et al., 2016), miR-27a-3p (Zhang et al., 2019), and miR-23a-3p (Rogler et al., 2017) have been previously shown to inhibit fibrotic diseases. Many studies have shown that these four miRNAs directly target the TGF-βR, and consistent with our results. Additionally, we found that miR-21-5p acts as an inhibitor of TGF-βR2 in the regulation of myofibroblast differentiation, which is in contrast to previous reports (Yuan et al., 2017). Generally, miRNAs can target different mRNAs at the same time. Therefore, we suggest that miR-21-5p may be a double-edged sword in regulating the TGF-β signaling pathway as its function may change according to the state of the cell and the particular molecular network involved. On the basis of the above results, we believe that hAFSC-exo-miRNAs may be important regulators of the TGF-β signaling pathway by inhibiting myofibroblast differentiation during cutaneous wound healing.

## Conclusion

**FIGURE 9:**
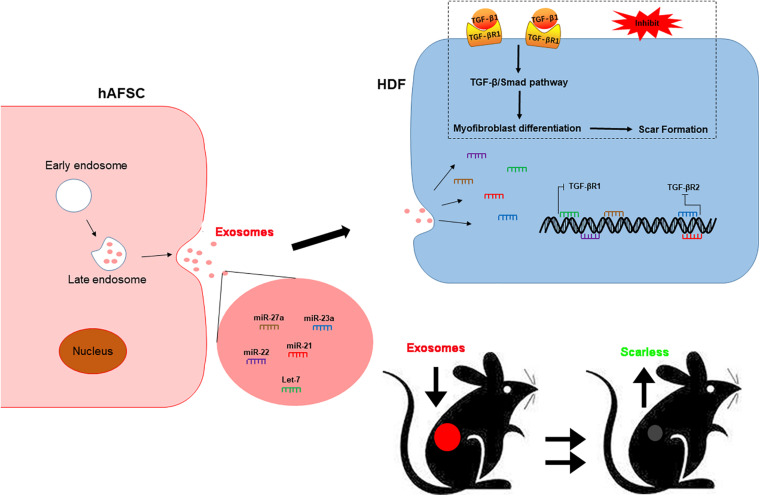
hAFSC-exo improves regeneration and suppresses fibrotic scarring by inhibiting the TGF-β signaling pathway. Let-7-5p, miR-22-3p, miR-27a-3p, miR-21-5p, and miR-23a-3p carried by hAFSC-exo may inhibit myofibroblast formation by down-regulating the expression of TGF-βR1 and TGF-βR2 in the TGF-β signaling pathway.

In conclusion, our study revealed that hAFSCs promote wound healing and prevent fibrotic scarring through exosomal specific miRNAs. Moreover, hAFSC-exo-specific-miRNAs inhibited myofibroblast formation and resulted in scarless wound healing by down-regulating TGF-βR1 by let-7-5p, miR-22-3p, and miR-27a-3p and TGF-βR2 by miR-21-5p and miR-23a-3p (Figure 9). As an alternative for cell therapy, hAFSC-exo may be benefit hair follicle regeneration and anti-scarring treatments in the clinic.

## Data Availability Statement

The original contributions presented in the study are included in the article/Supplementary Material, further inquiries can be directed to the corresponding author/s.

## Ethics Statement

The animal study was reviewed and approved by the Animal Experimental Ethics Committee of Jilin University (Approval No. SY201902011).

## Author Contributions

YZ: conception and design, collection of data, data analysis and interpretation, and manuscript writing. JY and YL: performance of the cell experiments, and collection and/or assembly of data. XL and LT: performance of the animal experiments, and collection and/or assembly of data. ZC and JL: collection and/or assembly of data. MD and JL: conception and design, data analysis and interpretation, financial support, and administrative support. GZ: conception and design, proofreading and manuscript writing, and final approval of the manuscript. All authors contributed to the article and approved the submitted version.

## Conflict of Interest

The authors declare that the research was conducted in the absence of any commercial or financial relationships that could be construed as a potential conflictof interest.

## Footnotes

1 http://www.targetscan.org/
