# Supplementary figures and images for: Human Amniotic Fluid Stem Cell-Derived Exosomes as a Novel Cell-Free Therapy for Cutaneous Regeneration
Source: Front Cell Dev Biol. 2021 Jun 21;9:685873. doi: 10.3389/fcell.2021.685873 (PMC8255501; doi:10.3389/fcell.2021.685873)

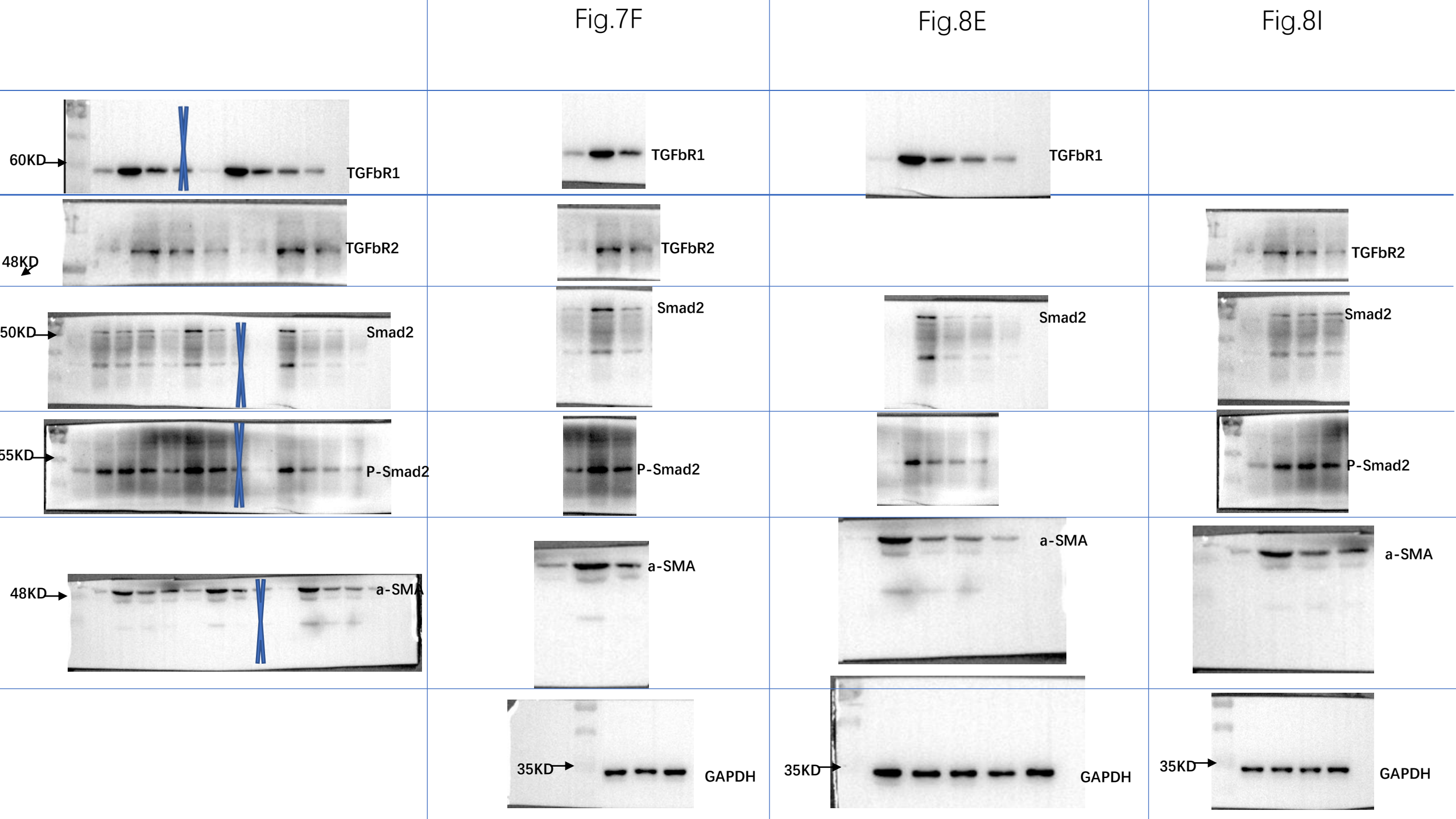

Supplement: Supplementary file 1 [file Image_1.PDF]
