# Supplementary material for: Human Amniotic Fluid Stem Cell-Derived Exosomes as a Novel Cell-Free Therapy for Cutaneous Regeneration
Source: Front Cell Dev Biol. 2021 Jun 21;9:685873. doi: 10.3389/fcell.2021.685873 (PMC8255501; doi:10.3389/fcell.2021.685873)
Supplement: Supplementary file 2 [file Table_1.docx]

Supplementary Material

**TABLE S1** Antibodies

| **Antibody** | **Company** | **Catalog number** | **Lot number** | **Dilution** |
| --- | --- | --- | --- | --- |
| anti-CD34 | Bioss, China | bs-0646R |  | IF (1:500) |
| anti-CD45 | Bioss, China | bs-4819R |  | IF (1:500) |
| anti-CD73 | Bioss, China | bs-4834R | AI10083001 | IF (1:500) |
| anti-CD90 | Bioss, China | bs-20640R |  | IF (1:500) |
| anti-CD105 | Bioss, China | bs-0579R |  | IF (1:500) |
| anti-CD31 | Bioss, China | bs-0468R | AF11255123 | IF (1:500) |
| anti-Nestin | Bioss, China | bs-0008R | AH04084807 | IF (1:500) |
| anti-Ki67 | Bioss, China | bs-23105R | AH07242946 | IF (1:500) |
| anti-α-SMA | Bioss, China | bsm-33188M | AG08161133 | IF (1:500), WB (1:1000 |
| anti-Smad2 | Beyotime, China | AF1300 | 090820201117 | WB (1:1000 |
| anti-p-Smad2 | Beyotime, China | AF5920 | 072720201217 | WB (1:1000 |
| anti-Collagen I | Bioss, China | bs-10423R | AH08315802 | IF (1:500) |
| anti-TGF-βR1 | Bioss, China | bs0638R | AD03051269 | IF (1:500), WB (1:1000 |
| anti-TGF-βR2 | Bioss, China | bs0117R | AI08095429 | IF (1:300), WB (1:1000 |

**TABLE S2** Primers

|  | | **qRT-PCR primers** |
| --- | --- | --- |
| α-SMA | F | AGCCATGTACGTAGCCATCC |
|  | R | CTCTCAGCTGTGGTGGTGAA |
| CD31 | F | AGTGTGGAAACCAACAGCCA |
|  | R | GCCTTCCGTTCTCTTGGTGA |
| Col1a2 | F | ggtgcccctggagagaat |
|  | R | ggaccagcagacccaatg |
| Col3a1 | F | gtccacgaggtgacaaaggt |
|  | R | catcttttccaggaggtcca |
| GAPDH | F | TGCCCCCATGTTTGTGATG |
|  | R | TGTGGTCATGAGCCCTTCC |
| Ki67 | F | GGACACCAGAGACCATGCAA |
|  | R | CCACGAGTTACCCTTGGCTT |
| MMP1 | F | gctttggcttccctagcagtg |
|  | R | tcgcctttttggaaaacatc |
| MMP3 | F | ccaccgagctatccactcat |
|  | R | gtccggtttcagcatgtttt |
| Nestin | F | TCGCTAGGGTCTGTGGATGA |
|  | R | GCTCTGTCACCTCTGGCATT |
| TGF-β1 | F | atacgcctgagtggctgtct |
|  | R | tgggactgatcccattgatt |
| TGF-β3 | F | ctctctgtccacttgcacca |
|  | R | tgcatctcttccagcaactcc |
| TGF-βR1 | F | TGCCTGCTTCTCATCGTGTT |
|  | R | TGCTTTTCTGTAGTTGGGAGT |
| TGF-βR2 | F | CTGCCCATCCACTGAGACATA |
|  | R | AGCTTGGGGTCATGGCAAAC |
| TIMP1 | F | catggagagcctctgtggat |
|  | R | atggctgaacagggaaacac |
| hsa-let-7-5p | F | ACACTCCAGCTGGGAGAGGTAGTAGGTTGC |
|  | R | CTCAACTGGTGTCGTGGAGTCGGCAATTCAGTTGAGAACTATGC |
| hsa-miR-22-3p | F | ACACTCCAGCTGGGAAGCTGCCAGTTGAAG |
|  | R | CTCAACTGGTGTCGTGGAGTCGGCAATTCAGTTGAGACAGTTCT |
| hsa-miR-27a-3p | F | ACACTCCAGCTGGGTTCACAGTGGCTAAG |
|  | R | CTCAACTGGTGTCGTGGAGTCGGCAATTCAGTTGAGGCGGAACT |
| hsa-miR-21-5p | F | ACACTCCAGCTGGGUAGCTTATCAGACTGA |
|  | R | CTCAACTGGTGTCGTGGAGTCGGCAATTCAGTTGAGTCAACATC |
| hsa-miR-23a-3p | F | ACACTCCAGCTGGGATCACATTGCCAGGG |
|  | R | CTCAACTGGTGTCGTGGAGTCGGCAATTCAGTTGAGGGAAATCC |
| U6 | F | CTCGCTTCGGCAGCACA |
|  | R | AACGCTTCACGAATTTGCGT |
